# Mulberry leaves juice attenuates arsenic‐induced neurobehavioral and hepatic disorders in mice

**DOI:** 10.1002/fsn3.3028

**Published:** 2022-08-17

**Authors:** Zohurul Islam, Jahidul Islam, Selim Reza Tony, Adiba Anjum, Rafia Ferdous, Apurba Kumar Roy, Shakhawoat Hossain, Kazi Abdus Salam, Farjana Nikkon, Khaled Hossain, Zahangir Alam Saud

**Affiliations:** ^1^ Department of Biochemistry and Molecular Biology University of Rajshahi Rajshahi Bangladesh; ^2^ Department of Biochemistry and Molecular Biology Mawlana Bhashani Science and Technology University Tangail Bangladesh; ^3^ Department of Pharmacy University of Rajshahi Rajshahi Bangladesh; ^4^ Department of Genetic Engineering & Biotechnology University of Rajshahi Rajshahi Bangladesh

**Keywords:** anxiety, arsenic exposure, learning and memory, oxidative stress

## Abstract

Arsenic (As) poisoning has caused an environmental catastrophe in Bangladesh as millions of people are exposed to As‐contaminated drinking water. Chronic As‐exposure causes depression, memory impairment, and liver injury in experimental animals. This study was carried out to assess the protective effect of mulberry leaves juice (Mul) against As‐induced neurobehavioral and hepatic dysfunctions in Swiss albino mice. As‐exposed mice spent significantly reduced time in open arms and increased time spent in closed arms in the elevated plus maze (EPM) test, whereas they took significantly longer time to find the hidden platform in the Morris water maze (MWM) test and spent significantly less time in the desired quadrant when compared to the control mice. A significant reduction in serum BChE activity, an indicator of As‐induced neurotoxicity‐associated behavioral changes, was noted in As‐exposed mice compared to control mice. Supplementation of Mul to As‐exposed mice significantly increased serum BChE activity, increased the time spent in open arms and reduced time latency to find the hidden platform, and stayed more time in the target quadrant in EPM and MWM tests, respectively, compared to As‐exposed‐only mice. Also, a significantly reduced activity of BChE, AChE, SOD, and GSH in brain, and elevated ALP, AST, and ALT activities in serum were noted in As‐exposed mice when compared to control mice. Mul supplementation significantly restored the activity of these enzymes and also recovered As‐induced alterations in hepatic tissue in As‐exposed mice. In conclusion, this study suggested that mulberry leaves juice attenuates As‐induced neurobehavioral and hepatic dysfunction in mice.

## Graphical abstract

Supplementation of mulberry leaves juice to As‐exposed mice significantly increased serum BChE activity, increased the time spent in open arms and reduced time latency to find the hidden platform and stayed more time in the target quadrant in EPM and MWM tests, respectively compared to As‐exposed only mice. Also, a significant reduced activity of BChE, AChE, SOD, and GSH in brain, and elevated ALP, AST and ALT activity in serum were noted in As‐exposed mice when compared to control mice.

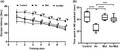

## 1 INTRODUCTION

Arsenic (As) contamination in groundwater is considered a serious public health problem in Bangladesh. Tube wells are the main source of drinking water in the rural areas of Bangladesh (WHO, Regional Office for South‐East Asia, 2002). As‐contaminated tube wells are common in the Northwest region of Bangladesh and about 16 million people are at risk of exposure to As by consuming tube‐wells water contaminated with As (Ali et al., 2010). As‐contaminated drinking water is the main source from which humans are exposed to As, thus, a significant portion of the population of Bangladesh suffers from As poisoning. Prolonged exposure to As through contaminated drinking water can cause acute and chronic toxic effects on all organs and systems, including skin, liver, nervous, cardiovascular, endocrine, and respiratory systems of the body (Mazumder et al., 1998; Parvez et al., 2006; Tapio & Grosche, 2006; Tyler & Allan, 2014; Wasserman et al., 2004; Rodríguez et al., 2002). Acute toxicity may develop for a short period of time, whereas chronic arsenicosis may take a longer time or even not manifest clinically in more than 10 years (Ahmad et al., 2018). The severity of As toxicity depends on various factors such as the chemical and physical form of As or its derivative compounds, the route of entry, the amount and duration of exposure, and the level of interacting factors in food, age, and sex of the individuals (Ratnaike, 2003). Generally, As is methylated and conjugated to glutathione, which is associated with the generation of oxidative stress both in humans and animals (Kumagai & Sumi, 2007; Thomas et al., 2004). More importantly, As can cross the blood–brain barrier, thus, can accumulate in various parts of the brain and lead to neurobehavioral abnormalities in both humans and laboratory animals (Prakash et al., 2016; Rodríguez et al., 2001; Sanchez‐Pena et al., 2010). For example, As exposure during the development period has been associated with neural tube abnormalities and anencephaly, as well as decreased motor activity and behavioral abnormalities in As‐exposed mice (Mazumdar et al., 2015: Wlodarczyk et al., 2006; Rodríguez, 2003). In addition, in previous studies, we have reported that chronic As exposure significantly impairs learning and memory function and leads to the development of anxiety‐like behavior in mice (Aktar et al., 2017; Biswas et al., 2020). The metabolic function of the liver is mainly to detoxify the toxins and carcinogens, and metal‐induced liver damage can cause acute hepatitis, cholestasis, and also develop liver cirrhosis (Singh et al., 2014). Liver is the primary target for As‐induced toxicity, where the highest amount of As accumulation was detected in the liver of the As‐exposed experimental animals (Singh et al., 2011). Exposure to As reportedly changed blood biochemical parameters, especially those related to liver disease in humans and experimental animals (Aktar et al., 2017; Ali et al., 2010).

Chronic As exposure is associated with several diseases, organ damage, and death; however, an effective treatment or management for As poisoning is yet to be established. Currently, there are some chelating agents that can bind to As from blood proteins, however, they are only used in the case of severe As toxicity (Bjorklund et al., 2020). Also, it is believed that these chelating agents can contribute to the development of many other diseases in the human body. Thus, finding alternatives for As poisoning as a protective resource would have great potential in mitigating the health problems, especially population highly exposed to As. Since plants are a rich source of biologically active compounds and provide medicinal products for the treatment of many diseases (Naowaratwattana et al., 2010), we are looking for a potential natural resource against As poisoning. In this study, we have explored *Morus alba* L., which is known worldwide as the black mulberry, and its fruit is also known for being low in calories and high in phytonutrients such as health‐promoting polyphenols and vitamins (Gundogdu et al., 2011). Mulberry has been used in traditional Chinese medicine as a tonic to treat and prevent diabetes and improve health. It also has potent antioxidant, anticancer, hyperlipidemic, and neuroprotective properties, and is used for the prevention of several chronic diseases (Jeong et al., 2010; Liu et al., 2009; Kim et al., 2010; Yang et al., 2010).

Mulberry leaves are a rich source of valuable bioactive compounds with significant nutraceutical values and antioxidant properties (Polumackanycz et al., 2021). The active ingredients of Mulberry leaves include chlorogenic acid, caffeic acid, quercetin glucoside, kaempferol glucoside, and quercetin rutin (Tchabo et al., 2018). It also contains various flavonoid compounds, which could scavenge the free radicals, thereby exhibiting a protective effect against many human diseases (Chan et al., 2016; Cui et al., 2019; Kim et al., 2014). Additionally, mulberry leaves have exhibited anti‐inflammatory, antinociceptive, and hepatoprotective effects (Malhi et al., 2014; Padilha et al., 2009, 2010). Recently, mulberry leaves are used as tea in many countries and are recognized as an excellent food resource due to their high content of protein, carbohydrates, vitamins, trace elements, and dietary fiber (Katsube et al., 2009; Polumackanycz et al., 2021). Also, oral administration of mulberry leaf extract is safe and nontoxic to the female reproductive system and embryonic development (Oliveira et al., 2013; Queiroz et al., 2012; Volpato et al., 2011). GABA depletion in the brain has been related to numerous neurological conditions such as Alzheimer's and Parkinson's diseases, and elevation of GABA accumulation followed by mulberry leaves treatment has been reported to work against cerebral ischemia both in vitro and in vivo (Kang et al., 2006). However, currently, there is no information on the protective effect of mulberry leave extract against As‐induced neurobehavioral and hepatic disorders. Therefore, the present study aimed to evaluate the protective effect of mulberry leaves juice against As‐induced anxiety‐like behavior, learning and memory impairment, as well as liver dysfunctions in mice.

## 2 MATERIALS AND METHODS

### 2.1 Collection and preparation of mulberry leaves juice (Mul)

Fresh young leaves of white mulberry plants were collected from Rajshahi University Campus, Rajshahi, Bangladesh, and recognized by a professional taxonomist at the Department of Botany, University of Rajshahi (voucher specimen no. RR 645). The leaves were washed thoroughly with distilled water and the petioles of each leaf were removed. After that 7500 mg of leaves without petioles were ground with a mortar pestle to form a paste, and then the leaves paste was dissolved in double‐distilled water to make 500 ml, and finally, the concentration of leaves juice was 1.5 mg/ml. Mulberry leaves juice was kept at 4°C in a sealed glass bottle to avoid microbial contamination until use.

### 2.2 Animal maintenance and experimental design

Swiss Albino male mice of 4 weeks aged were divided into four groups: (a) control group, (b) arsenic group (As), (c) mulberry leaves juice group (Mul), and (d) arsenic plus mulberry leave group (As+Mul). Sodium arsenite (NaAsO_2_) was given to As and As+Mul group mice through intraperitoneal injection (10 mg/kg body weight) 1‐day interval for 30 days, and mice from the control and Mul group received physiological saline only as a vehicle. Mice of Mul and As+Mul group were treated with mulberry leaves juice (50 mg/kg body weight) and the juice was given to the stomach of the mice through an oral gavage tube once a day all through the experimental periods. However, mice from the control and As‐treated group received distilled water at the same time as a vehicle. Mice of each group (10 mice) were kept in each cage and were maintained at 25 ± 2°C with natural light and dark cycle as reported in Rashid et al. (2017). All the experimental mice from four groups were treated for 30 days as stated above and fed by normal food ad libitum. Ethical authorization of the experimental protocol was received from the Institute of Biological Sciences, University of Rajshahi, Bangladesh (No: 255[14]/320/IAMEBBC/IBSc).

### 2.3 Anxiety assessment in the elevated plus maze (EPM)

EPM is an extensively used tool to assess anxiety‐like behavior in laboratory animals. The EPM consists of two open arms of 50 × 10 cm (length and width), two closed arms of 50 × 10 × 40 cm (length, width, and height), and the height of arms was 50 cm above the ground, as described by Aktar et al. (2017). Initially, each mouse was independently placed in the middle of the stage facing one of the closed arms according to the method described by Koehl et al. (2001). All of the four paws of the animal in the particular arms were reflected as an effective access into the closed arms, and the comparison of time staying in open arms and closed arms was considered anxiety‐liked behavior (Schneider et al., 2011). After each test, a 70% ethanol solution was used to clean the maze and to eliminate the effect of outcomes due to olfactory hints.

### 2.4 Spatial memory and learning assessment in the Morris water maze

The maze was hypothetically divided into four quadrants of equal size, and a platform was positioned and submerged the water surface in the middle of the northeast (NE) quadrant of the pool as described previously (Aktar et al., 2017). Mice have been training for 2 days to recall the place of the platform, and a maximum of 60 seconds were given to each animal to discover the invisible platform and allowed to stay on the platform for 20 seconds. Mice individuals who could not find the submerged platform within the set time have been guided to the platform and allowed to stay on the platform for 20 s with the guidance of an experimenter. Every day, three trials have been conducted, and approximately 30 min were given as an intertrial interval as described by Barnhart et al. (2015). After 2 days of training, the experiment was conducted for 7 days, and the time required to discover the platform for each trial was documented. The average time of the three trials reflected the animal's escape latency each day, and the decrease in the escape latency compared to the previous day was expressed as complete learning. At the end of each trial, the mice were dried with a towel and kept back in a warming cage. After the 7‐day trials, a probe test was performed without the platform in the maze, and the time spent in the NE quadrant was documented within the 60 sec as reported previously (Rashid et al., 2017). The mouse stayed more time in the desired quadrant during the probe test indicating better performance.

### 2.5 Blood biochemical analysis of serum

One week after the behavioral tests, the experimental animals were sacrificed. After anesthesia with diethyl ether, blood samples were taken from the thoracic artery of the experimental mice. The serum was collected from the blood samples and deposited at −80°C before the biochemical assay as described in Biswas et al. (2019). Butyrylcholinesterase (BChE) activity in serum was measured using the analyzer according to the manufacturer's instructions (RANDOX, UK). Kits from Human Diagnostic (Germany) were used to measure the enzymatic activities of aspartate aminotransferase (AST), alanine aminotransferase (ALT), and alkaline phosphatase (ALP) in the serum using an analyzer (Humalyzer‐3000, Germany).

### 2.6 Biochemical analysis from brain tissue homogenates

The whole brains were isolated, cleaned, blotted, and weighed using an electronic balance and homogenized in phosphate buffer (0.1 M, pH 7) containing 0.5% Triton X100 (Sigma 318K, Germany), followed by centrifugation at 5000 rpm at 4°C, and then the supernatant was subjected to various biochemical evaluations as described previously (Reza et al., 2018). The activity of superoxide dismutase (SOD) and glutathione (GSH) in brain tissue homogenate of experimental mice was measured according to the protocols mentioned earlier (Ellman, 1959; Marklund & Marklund, 1974). The total amount of protein was estimated using the Lowry method (Lowry et al., 1951). The activity of acetylcholinesterase (AChE) and butyrylcholinesterase (BChE) in the brain tissue homogenate was measured as described previously (Ellman et al., 1961). The average values of the two tests were used to analyze each sample. Mice that did not achieve the learning criteria were excluded from biochemical analysis.

### 2.7 Histopathological study of hepatic tissues

The livers of the experimental mice were carefully washed with saline water and preserved in 10% formalin solution (10%) to fix the organ. Ethanol was used to dehydrate the samples and then embedded in paraffin. Microtome was used to obtain 5‐μm‐thick section from paraffin‐embedded tissue samples. Tissue sections were stained using the conventional pathological technique as stated previously (Biswas et al., 2019) and tissue slices were examined under a light microscope.

### 2.8 Statistical analysis

Data were expressed as median (range) values or mean ± SEM (standard error of mean) as stated. The statistical significance among the experimental groups was assessed using Welch's test and ordinary one‐way ANOVA as directed. *p* values <.05 were considered statistically significant. Data were analyzed and graphs were created with GraphPad Prism 7.05 (GraphPad Software, La Jolla, CA).

## 3 RESULTS

### 3.1 Impact of Mul against As‐induced anxiety‐like behavior in mice

**FIGURE 1:**
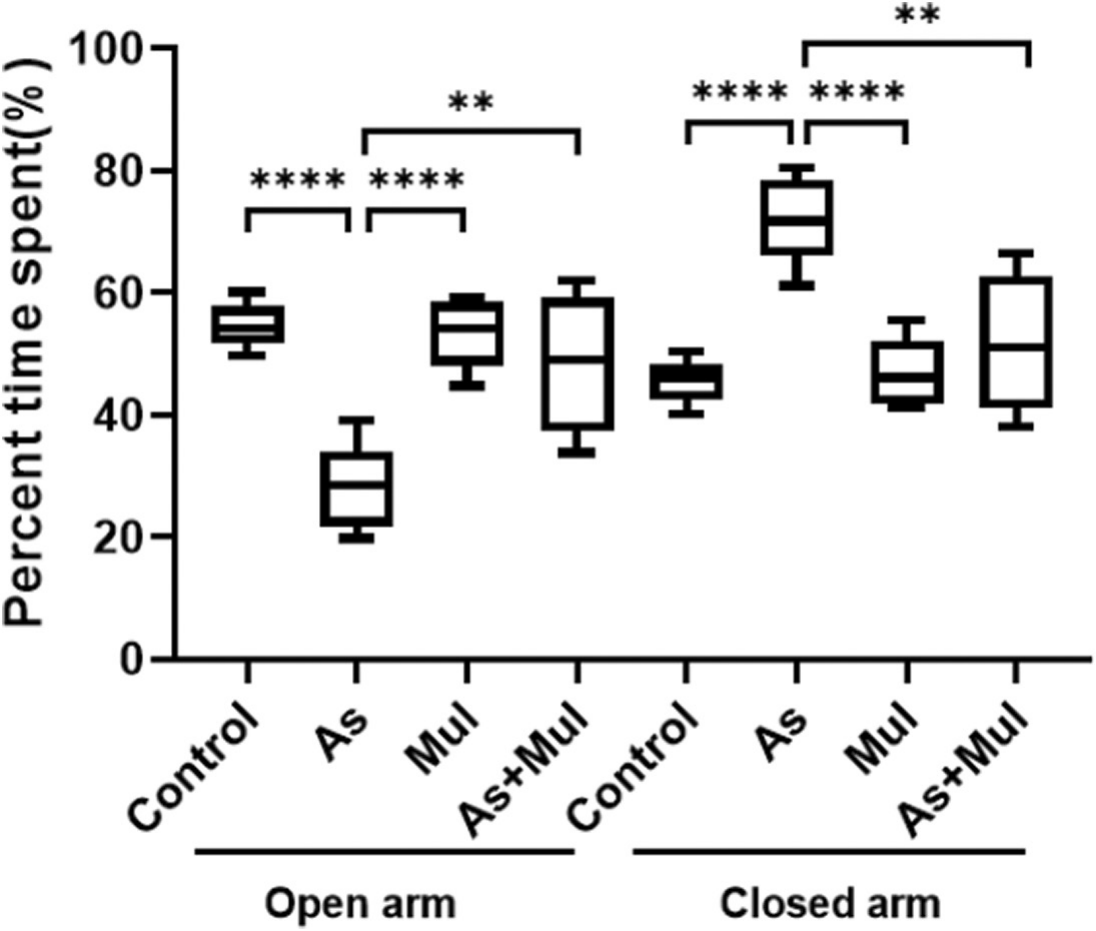
Percent time spent in open and closed arms of experimental mice in an elevated plus maze. Box plots indicate the time spent in open and closed arms of control, arsenic (As), mulberry (Mul), and arsenic plus mulberry (As+Mul) mice. Data were expressed as median (range) values, where *n* = 7 for each group of mice. The upper and lower limits of the boxes and the middle line across the boxes indicate the maximum, minimum, and median values, respectively. Group comparisons were performed by Welch's *t*‐test (**p < .01, ****p < .0001) and ordinary one‐way ANOVA (*p* < .0001)

The results of the EPM test on experimental mice are shown in Figure 1. The percentage of time spent within the open arms or closed arms was chosen for the EPM anxiety index. The percentages of time spent in open arms of control, As, Mul, and As+Mul group mice were 54.333 ± 1.321, 29.000 ± 2.623, 52.666 ± 2.109, and 48.619 ± 4.034 (Mean ± SEM), respectively, while the percentages of time spent in closed arms of the four groups were 45.667 ± 1.321, 71.000 ± 2.623, 47.334 ± 2.109, and 51.381 ± 4.034, respectively. Results showed that As exposure significantly (*p* < .0001) reduced the time spent in open arms, whereas increased the time spent in closed arms as compared to control mice. However, supplementation of mulberry leaves juice increased the time spent in open arms of As+Mul mice (*p* < .01) compared to As‐treated mice.

### 3.2 Protective effect of Mul against As‐treated spatial memory and learning impairment

**FIGURE 2:**
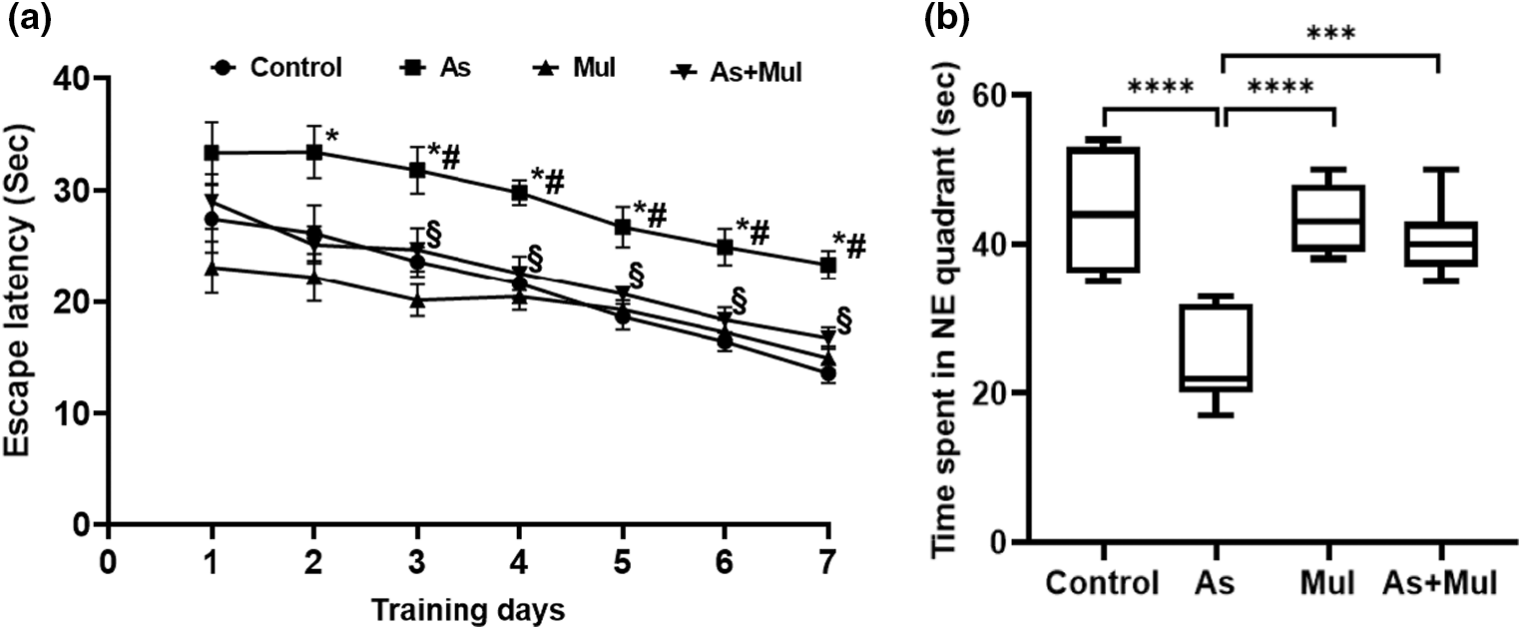
Protective effects of Mul on As‐induced learning and memory impairment of mice in MWM. (a) Latency time of control, arsenic (As), mulberry (Mul), and arsenic plus mulberry (As+ Mul) were expressed as Mean ± SEM, where *n* = 7 for each group of mice. Mice were trained in three trials per day. ^*^Significantly different from the control group at *p* < .05 in Welch's *t*‐test. ^#^Significantly different from the control group at *p* < .05 in ordinary one‐way ANOVA. ^§^Significantly different from the Arsenic (As) group at *p* < .05 in both Welch's *t*‐test and ordinary one‐way ANOVA. (b) MWM probe test for experimental mice. Time spent in the NE quadrant of control, arsenic (As), mulberry (Mul), and arsenic plus mulberry (As+Mul) mice groups were expressed as median (range) values, where *n* = 7 for each group of mice. The boxes and bars are as in Figure 1. Group comparisons were performed by Welch's *t*‐test (***p = .0002, ****p ˂ .0001) and ordinary one‐way ANOVA (*p* < .0001)

Protective effects of Mul against As‐induced spatial memory and learning impairment in mice are depicted in Figure 2. The results demonstrated the escape latency to reach the platform. On the initial day of spatial acquisition trials, all mice had a tendency to swim around the edges to escape. However, when being placed on the platform at the end of every trial, mice learned step by step that there was an escape platform into the pool. In the Morris water maze test, the mean latency time (Mean ± SEM) of the control group to find the platform was 27.48 ± 3.013 s on the 1st day, which decreased rapidly during the learning for 7 days and the mean latency time was 13.59 ± 0.700 s on the 7th day. Whereas the mean latency time of As‐treated, Mul, and As+Mul group mice were 33.36 ± 2.740, 23.12 ± 2.332, and 29.02 ± 2.444 s on day 1, respectively, and 23.36 ± 1.228, 14.93 ± 1.078, and 16.81 ± 1.015 s, respectively, on day 7. The results showed that the mean latency time of As‐treated mice was significantly higher (*p* < .0001) and slightly reduced during 7 days of learning as compared to the control group. However, supplementation of Mul to As‐treated mice significantly reduced the mean latency time compared to As‐treated mice (*p* < .001), which indicated an improvement of learning capability during 7 days of training as compared to the As‐treated mice group [for day 3, *F*_(3, 24)_ = 8.058 *p* = .0007; for day 4, *F*_(3, 24)_ = 8.614, *p* = .0005; for day 5, *F*_(3, 24)_ = 9.974, *p* = .0002; for day 6, *F*_(3, 24)_ = 10.32, *p* = .0001; and for day 7, *F*_(3, 24)_ = 17.89, *p* < .0001]. We also carried out a probe trail to verify the learning and spatial memory on day 8 in the maze without the platform. During the 60 sec experiments, the time spent in the desired quadrant of the control, As‐treated, Mul, and As+Mul groups were 45.140 ± 3.027, 24.290 ± 2.275, 43.860 ± 1.818, and 40.860 ± 1.844 s, respectively (Figure 2b). The results of this study showed that time spent in the desired quadrant of control mice was significantly higher than that of As‐treated mice (*p* < .001). In addition, the result of this study also demonstrated that As‐treated mice supplemented with mulberry leaves juice stayed significantly more time in the desired quadrant compared to As‐treated mice (*p* < 0.001).

### 3.3 Effect of Mul juice on serum BChE, ALP, AST, and ALT levels in experimental mice

**FIGURE 3:**
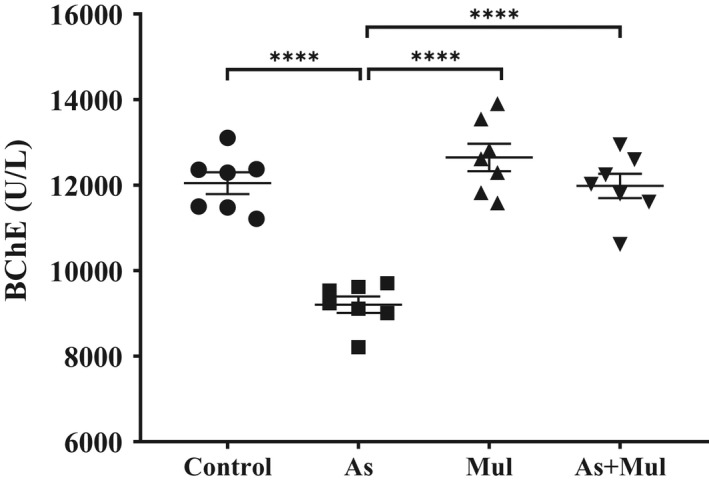
Serum BChE activity in experimental mice. Control, arsenic (As), mulberry (Mul), and arsenic plus mulberry (As+Mul) mice were expressed as mean ± SEM, where *n* = 7 for each group of mice. Significantly different means were performed by Welch's *t*‐test (****p < .0001) and ordinary one‐way ANOVA (*p* < .0001)

Blood biomarkers are useful for assessing the risk of hazardous metals, which interfere with the activity of numerous intracellular biological influences. The reduction in serum BChE activity reflects metal‐induced neurotoxicity and association with memory impairment in animals and humans (Anjum et al., 2019; Dong et al., 2017). Serum BChE activity of control, As‐treated, Mul, and As+Mul mice groups were 12045.000 ± 253.677, 9203.514 ± 192.824, 12647.071 ± 321.410, and 11980.714 ± 285.352 U/L, respectively (Figure 3). As‐exposure significantly reduced the BChE activity in serum compared to control mice (*p* < .0001). However, mulberry leaves juice supplementation significantly increased the BChE activity in As‐exposed mice (*p* < .0001).

The levels of liver enzymes in serum are elevated in individuals, who are chronically exposed to toxic substances. To explore the hepatoprotective effect of Mul on As‐exposed mice, we measured the levels of ALP, AST, and ALT in the serum of the experimental mice (Table 1). The ALP levels were 138.81 ± 4.76, 196.02 ± 6.71, 135.91 ± 2.43, and 154.14 ± 7.71 U/L in control, As, Mul, and As+Mul mice groups, respectively. The ALT levels for control, As, Mul, and As+Mul mice groups were 68.4 ± 3.17, 92.29 ± 3.71, 64.97 ± 3.85, and 70.13 ± 1.83 U/L, respectively, whereas AST levels were 58.59 ± 3.25, 81.93 ± 2.09, 57.33 ± 2.07, and 61.50 ± 2.25 U/L in c control, As, Mul, and As+Mul mice, respectively. Data showed that As exposure significantly increased serum ALP, ALT, and AST levels compared to control mice (*p* < .0001), however, supplementation of Mul in As‐exposure mice significantly reduced or restored the activity of the above‐mentioned enzymes compared to only As‐exposure mice groups (*p* < .001).

**TABLE 1:** Serum ALP, ALT, and AST activities of the experimental mice

Serum indices (U/L)	Experimental groups				*p*‐value
	Control	As	Mul	As+Mul	
ALP	138.81 ± 4.76	196.02 ± 5.47^a^	135.91 ± 2.43^b^	154.14 ± 2.33^c^	^a^ *p* < .0001, ^b^ *p* < .0001, ^c^ *p* = .0015
ALT	68.4 ± 3.17	92.29 ± 3.71^a^	64.97 ± 3.85^b^	70.13 ± 1.83^c^	^a^ *p* < .0004, ^b^ *p* = .0003, ^c^ *p* = .0005
AST	58.59 ± 3.25	81.93 ± 2.09^a^	57.33 ± 2.07^b^	61.50 ± 2.25^c^	^a^ *p* = .0001, ^b^ *p* < .0001, ^c^ *p* < .0001

*Note:* Data are expressed as Mean ± SEM, *n* = 7 for each group of mice. ^a^Significantly different from control, and ^b,c^significantly different from Arsenic (As) mice group. Group comparisons were performed by Welch's *t*‐test and ordinary one‐way ANOVA (*p* < .0001).

### 3.4 Effects of Mul against brain enzymes activity in As‐induced mice

In order to assess whether Mul can attenuate the neurotransmitter system destruction, we determined the activities of AChE and BChE in the brain of experimental mice (Table 2). The result showed that brain AChE activities were 133.56 ± 1.24, 116.26 ± 1.40, 136.96 ± 2.13, and 128.13 ± 2.09 mU/mg in control, As‐treated, Mul, and As+Mul group mice, respectively. On the other hand, BChE activities were 8.63 ± 0.40, 5.81 ± 0.34, 8.96 ± 0.31, and 7.51 ± 0.20 mU/mg in control, As‐treated, Mul, and As+Mul group mice, respectively. Results showed that As exposure promoted a significant reduction in both AChE and BChE levels (*p* < .001) compared to control mice. Concomitant treatment with Mul significantly restored (*p* < .001) the activities of AChE and BChE in the As‐exposure group.

**TABLE 2:** Brain AChE, BChE, GSH, and SOD activities of the experimental mice

Enzyme activity	Experimental groups				*p*‐value
	Control	As	Mul	As+Mul	
AChE (mU/mg)	133.56 ± 1.24	116.26 ± 1.40^a^	136.96 ± 2.13^b^	128.13 ± 2.09^c^	^a^ *p* < .0001, ^b^ *p* < .0001, ^c^ *p* = .0007
BChE (mU/mg)	8.63 ± 0.40	5.81 ± 0.34^a^	8.96 ± 0.31^b^	7.51 ± 0.20^c^	^a^ *p* = .0002, ^b^ *p* < .0001, ^c^ *p* = .0017
GSH (umol/mg)	0.228 ± 0.013	0.131 ± 0.004^a^	0.238 ± 0.006^b^	0.200 ± 0.016^c^	^a^ *p* = .0006, ^b^ *p* < .0001, ^c^ *p* = .0051
SOD (U/mg)	8.59 ± 0.25	5.35 ± 0.27^a^	8.76 ± 0.34^b^	7.85 ± 0.34^c^	^a^ *p* < .0001, ^b^ *p* < .0001, ^c^ *p* = .0001

*Note:* Data are expressed as Mean ± SE, *n* = 7 for each group of mice. ^a^Significantly different from control, and ^b,c^significantly different from Arsenic (As) mice group. Group comparisons were performed by Welch's *t*‐test and ordinary one‐way ANOVA (*p* < .0001).

The antioxidant enzymes SOD and GSH activities in the brain of the experimental mice group were presented in Table 2. The SOD activity of control, As‐treated, Mul, and As+Mul group mice were 8.59 ± 0.25, 5.35 ± 0.27, 8.76 ± 0.34, and 7.85 ± 0.34 U/mg, respectively. Statistical analysis showed that SOD activity reduced significantly in the brain of As‐exposure mice compared to control mice (*p* < .0001), however, supplementation of mulberry leaves juice significantly attenuated the reduction in the SOD activity in As+Mul mice compared to As‐treated mice (*p* < .001). The GSH activity in the brain tissues of control, As‐treated, Mul, and As+Mul groups mice were found as 0.228 ± 0.013, 0.131 ± 0.004, 0.238 ± 0.006, and 0.200 ± 0.016 umol/mg, respectively. GSH activity was found to be significantly reduced in As‐treated mice compared to control mice (*p* < .001), while GSH activity was significantly recovered in Mul‐supplemented As+Mul‐treated group of mice compared to As‐treated group (*p* < .01).

### 3.5 Effects of Mul on As‐induced histopathological alterations of the liver tissues

**FIGURE 4:**
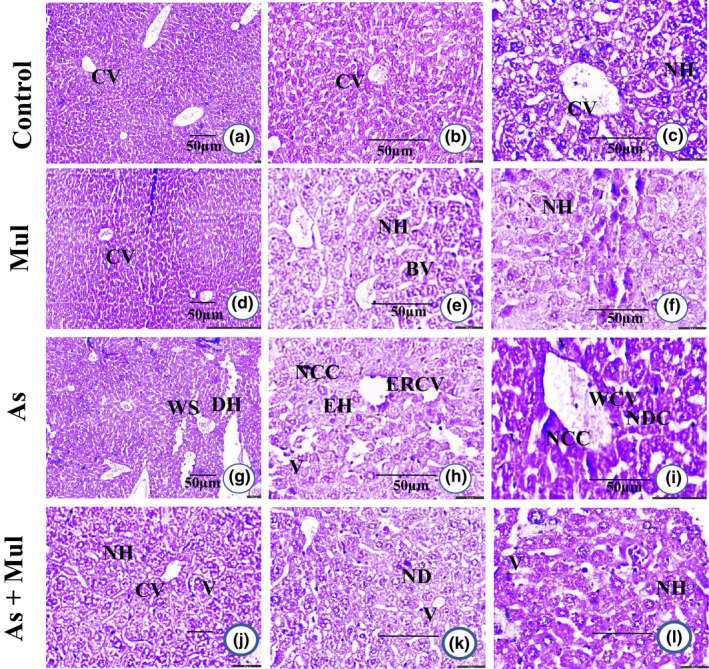
Histological photograph of hepatic tissue of experimental mice (H & E stain). Hepatic tissues of control mice (a, b and c) showed normal hepatocyte (NH) with the central vein (CV). Mul‐treated mice liver (d, e and f) also showed normal hepatocyte (NH) with the central vein (CV) and blood vessels (BV). Hepatic tissue section of As‐treated mice (g, h and i) showed widened central vein (WCV), endothelial rupture in the central vein (ERCV), widened sinusoidal space (WS), enlarged hepatocyte (EH), nuclear degenerative changes (NDC), the appearance of severe necrosis in the cytoplasm (NCC), appearance in vacuoles (V) endothelial rupture in the central vein (ERCV), and degenerated hepatocytes (DH). Mul coadministration with As (As+Mul) (j, k and l) showed moderate changes with the central vein (CV), normal hepatocyte (NH), few appearances in vacuoles (V), and nuclear degeneration (ND). Microphotography of a, d, g and j in ×10; b, e, h, and k in ×20; and others in ×40 with hematoxylin and eosin stain

Histopathological studies confirmed the regular hepatocytes structure with separated hepatic cells, sinusoidal areas in the hepatic tissues of control mice, and no histopathologic alterations were observed in Mul‐treated mice (Figure 4a–f). In the As‐treated group, loss of cellular structures such as swelling of blood sinusoids, degeneration of hepatocytes with dense nuclei, and lymphocyte aggregation in vacuolar cytoplasm in liver tissue was observed (Figure 4g–i). However, As‐induced pathological alterations were remarkedly improved with Mul supplementations found in the hepatic tissues of As+Mul‐treated mice (Figure 4j–l).

## 4 DISCUSSION

Reduction in antioxidant enzyme activity and elevation of reactive oxygen species (ROS) into brain tissue indicated brain damage in As‐exposed mice. As‐induced interruption of the antioxidant defense system is one of the major causes of memory impairment and anxiety‐like behavior development in experimental animals. These days, plant materials/herbs are a better option to prevent the development of metal‐induced toxicity, as plant‐based products have no side effects. Treatment of metal toxicity with plant materials or compounds derived from plants may be a better replacement for synthetic drugs. The results of the present study showed that mulberry leaves juice facilitated the cellular antioxidant defense system and also attenuated the adverse effects of As on the neurotransmitter system in the brain of As‐exposed mice. Several reports confirmed that As exposure induces neurotoxic effects such as the development of anxiety‐like behavior and learning and memory impairments in laboratory animals (Aktar et al., 2017; Rodríguez, 2003; Sanchez‐Pena et al., 2010). In our previous studies, we have noted that As‐exposed mice stayed longer time in the closed arm in the EPM test and also had less competence to recover as well as recall the place of the secreted platform even after 7 days of trial in MWM test compared to control mice (Aktar et al., 2017; Biswas et al., 2019). The finding of the present study is consistent with our previous studies. Importantly, current results confirmed that coadministration of Mul reduced the effects of As‐induced memory deficits and also reduced the development of anxiety‐like behavior in As‐treated mice. As exposure affects the activity of antioxidant enzymes and increases the oxidative stress in the brain of As‐exposed animals, and high oxidative stress is linked with the development of anxiety‐like behavior and depression (Allam et al., 2013; Patki et al., 2013). Also, both in vivo and in vitro studies suggest that chronic As exposure may interfere with neurotransmitters associated with depression and promotes oxidative stress, an impending mechanism of As‐induced neurotoxicity, which in turn, leads to abnormal brain development and behavioral disorders (Brinkel et al., 2009; Gandhi & Kumar, 2013).

The reduction in cholinesterase activity is associated with As exposure in the blood as well as in the brain (Ali et al., 2010; Anjum et al., 2019; Patlolla & Tchounwou, 2005). In the present study, we have found that Mul supplementation significantly restored the cholinesterase activities in As‐exposed mice. Mulberry leaves have potent antioxidant properties and play an effective role in the antioxidant defense system (Aramwit et al., 2013). We have noted that supplementation of mulberry leaves juice provided a protective role against As‐induced generation of oxidative stress in the brain of experimental mice and may eventually prevent the reduction in cholinesterase activity. In this study, we observed that AChE and BChE levels in the brain and BChE activity in serum decreased significantly in As‐treated mice when compared to that of control mice, whereas a significant recovery of both AChE and BChE activity was recorded in Mul‐supplemented As‐treated mice in comparison to that of As‐exposed mice. Several studies confirmed that As‐exposure affects cholinergic function, and cholinergic dysfunction has been associated with decreased brain cholinesterase activity and impaired learning and memory in laboratory animals (Flora et al., 2009; Rodríguez, 2003; Rodríguez et al., 2001; Yadav et al., 2011). Previous studies reported that the expression of brain cholinesterase plays an important role in cognitive function and a decreased cholinesterase activity is associated with inflammation, oxidative stress, and the development of neurotoxicity (Bono et al., 2015; Darvesh, 2016; Lockridge, 2015; Santarpia et al., 2013).

Oxidative stress refers to the uneven formation of ROS and the ability to rapidly detoxify reactive intermediates or repair damage to biological systems (Sinha et al., 2013). ROS plays an important role in As‐induced oxidative stress that persuades various pathophysiological conditions in experimental animals, and the reduced activity of antioxidant enzymes such as SOD and GSH in brain tissue of As‐exposure mice may cause oxidative damage in the brain and other deposited organs (Das et al., 2009; Sharma et al., 2010). Like other antioxidant enzymes, SOD and GSH play vital roles in neutralizing ROS and aggregated As methylation for cellular uptake (Vahter, 2002). Significant reductions in SOD and GSH activity were found in brain tissue of As‐exposed laboratory animals. Interestingly, the supplementation of Mul to As‐exposed mice could prevent As‐induced pathophysiology as well as re‐establish (i.e., restore) the level of antioxidant enzyme activity in the brain. Data analysis from the neurotransmitters system and the activity of antioxidant enzymes in brain tissue related to the neurobehavioral assays implied that Mul showed a unique ability to detoxify ROS. Thus, the significant protective effect of mulberry leaves might be due to the presence of higher concentrated active compounds, including alkaloids, phenolic acids, and flavonoids such as quercetin, kaempferol, chlorogenic acid, and rutin (Ju et al., 2018; Tchabo et al., 2018). It was noted that quercetin glucosides, a kind of flavonoid, exert therapeutic effects on animal models of neurodegeneration or neurotoxicity by inhibiting the fibrillar formation of Aβ protein, interfering with cellular lysis and inflammatory cascades (Aliev et al., 2008; Belo et al., 2013; Davis et al., 2009). Also, quercetin glucoside has the ability to reduce lipid peroxidation and ultimately protect the oxidative damage of neurons, thereby improving learning, memory, and cognitive functions via activating AMPK activity and decreasing mitochondrial dysfunction in animal models (Sabogal‐Guaqueta et al., 2015; Singh et al., 2016; Wang et al., 2014). Furthermore, antioxidant activity containing various natural compounds including quercetin glucosides can restore brain AChE activity in metal‐exposed experimental animals (Abdalla et al., 2013; Gonçalves et al., 2010; Schmatz et al., 2009; Gutierres et al., 2012). Therefore, it is assumed that active compounds present in supplemented Mul prevented the alterations of AChE activity in the brain tissue of As‐exposed mice and also could modulate cholinergic neurotransmission and enhance cognitive abilities.

The liver is one of the principal organs for removing toxic substances from the body, and levels of ALT, AST, and ALP are good indicators of hepatic function assessment (Biswas et al., 2019; Piñeiro‐Carrero & Piñeiro, 2004). In this study, we found that the levels of liver enzymes are elevated, however, supplementation of Mul significantly restored all of three enzymes in As‐exposed mice (Table 1). Histopathological observations also confirmed that Mul recovered As‐induced alteration in the hepatic tissues (Figure 4). Therefore, altogether, Mul provided protective functionality against As‐induced neuro‐ and hepatic toxicity by enhancing antioxidant properties. Active compounds present in the supplementation of Mul can make plausible binding and modify the activities of the antioxidant enzymes. However, further study is required to elucidate the exact molecular mechanisms of responsible compound(s) present in mulberry leaves against As‐induced neuro‐ and hepatic toxicity.

## 5 CONCLUSION

In conclusion, supplementation of mulberry leaves juice protected from the development of As‐induced anxiety‐like behavior and memory impairment as well as hepatic dysfunctions in As‐exposed experimental animals. The present study showed that anti‐inflammatory and antioxidant compounds present in mulberry leaves suppressed As‐induced toxicity by enhancing the antioxidant enzymes activity and neurochemical system in the brain of As exposed mice.

## CONFLICT OF INTEREST

The authors declare that there is no conflict of interest.
